# Chemotherapy-Induced Cutaneous Sclerosis: A Rare Clinical Finding

**DOI:** 10.7759/cureus.69870

**Published:** 2024-09-21

**Authors:** Duarte Flor, Joana a Calvão, Edgar Pratas, Inês Coutinho, Jose C Cardoso

**Affiliations:** 1 Dermatology, Coimbra Local Health Unit, Coimbra, PRT; 2 Oncology, Coimbra Local Health Unit, Coimbra, PRT; 3 Dermatology, University Hospital of Coimbra, Coimbra, PRT

**Keywords:** adverse reaction, chemotherapy, cutaneous sclerosis, nab-paclitaxel, pancreatic adenocarcinoma treatment

## Abstract

Scleroderma-like cutaneous sclerosis has been reported as a rare adverse reaction to several drugs, including the chemotherapeutical agent paclitaxel, used in therapeutic regimens for several malignancies. The sclerosis is usually limited to the skin, most commonly presenting in the lower limbs after weeks to months of therapy but is often refractory to treatment and progresses even after discontinuation of the offending agents, with significant resulting morbidity.

We report a rare case of severe cutaneous sclerosis secondary to chemotherapy with nab-paclitaxel and gemcitabine, which did not respond to treatment and led to discontinuation of chemotherapy.

## Introduction

Scleroderma-like cutaneous sclerosis has been reported as a rare adverse reaction to several drugs, particularly the chemotherapeutical agent paclitaxel [1]. Gemcitabine and paclitaxel, used as a chemotherapeutical regimen for pancreatic adenocarcinoma, have also been associated with neutropenia, anemia, and peripheral neuropathy [2]. The incidence, physiopathology, and risk factors for the reaction are poorly established. Clinical features include cutaneous sclerosis and edema, most prominent in the lower limbs, usually starting weeks to a few months after initiation of therapy [1]. The sclerosis is usually cutaneous rather than systemic (as opposed to diseases such as systemic scleroderma), but is often refractory to treatment and progresses even after discontinuation of the offending agents, with significant resulting morbidity [1,3-7].

We report a case of cutaneous sclerosis secondary to chemotherapy with nab-paclitaxel and gemcitabine.

## Case presentation

A 68-year-old male with prior diabetes stage IV pancreatic adenocarcinoma with extensive peritoneal carcinomatosis was referred to dermatology due to cutaneous sclerosis and edema of the extremities, most prominent in the lower limbs. He had completed 19 cycles of nab-paclitaxel and gemcitabine (every three weeks), which he had started around 15 months prior as first-line, palliative chemotherapy.

**Figure 1:**
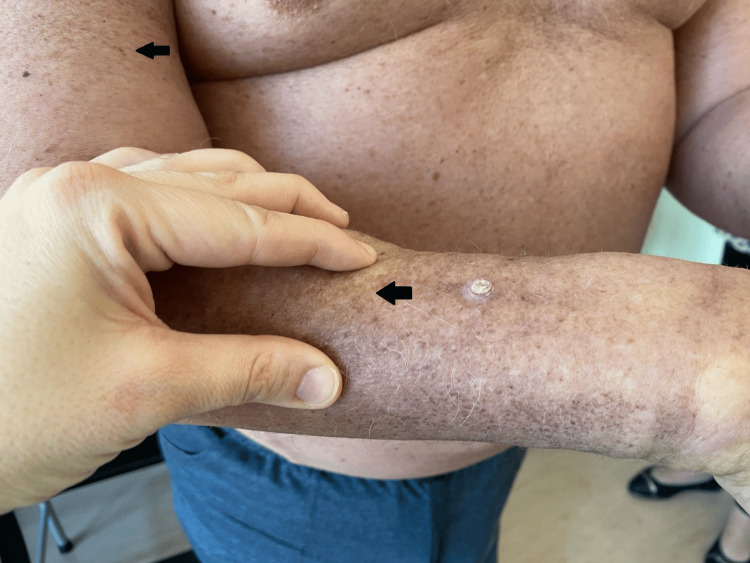
Cutaneous thickening with coarse, sclerotic surface, and superficial scales (black arrows)

He presented widespread and marked cutaneous thickening with a coarse, sclerotic surface, with slight erythema and superficial scaling (Figure 1). The findings were most prominent in the limbs but also present in the trunk. Raynaud phenomena were absent. Antinuclear antibodies were negative, and the remaining blood analysis and urinalysis were unremarkable. Chest, abdominal, and pelvic CT showed disease stability since onset, with no increase in tumor burden and no other significant changes. 

**Figure 2:**
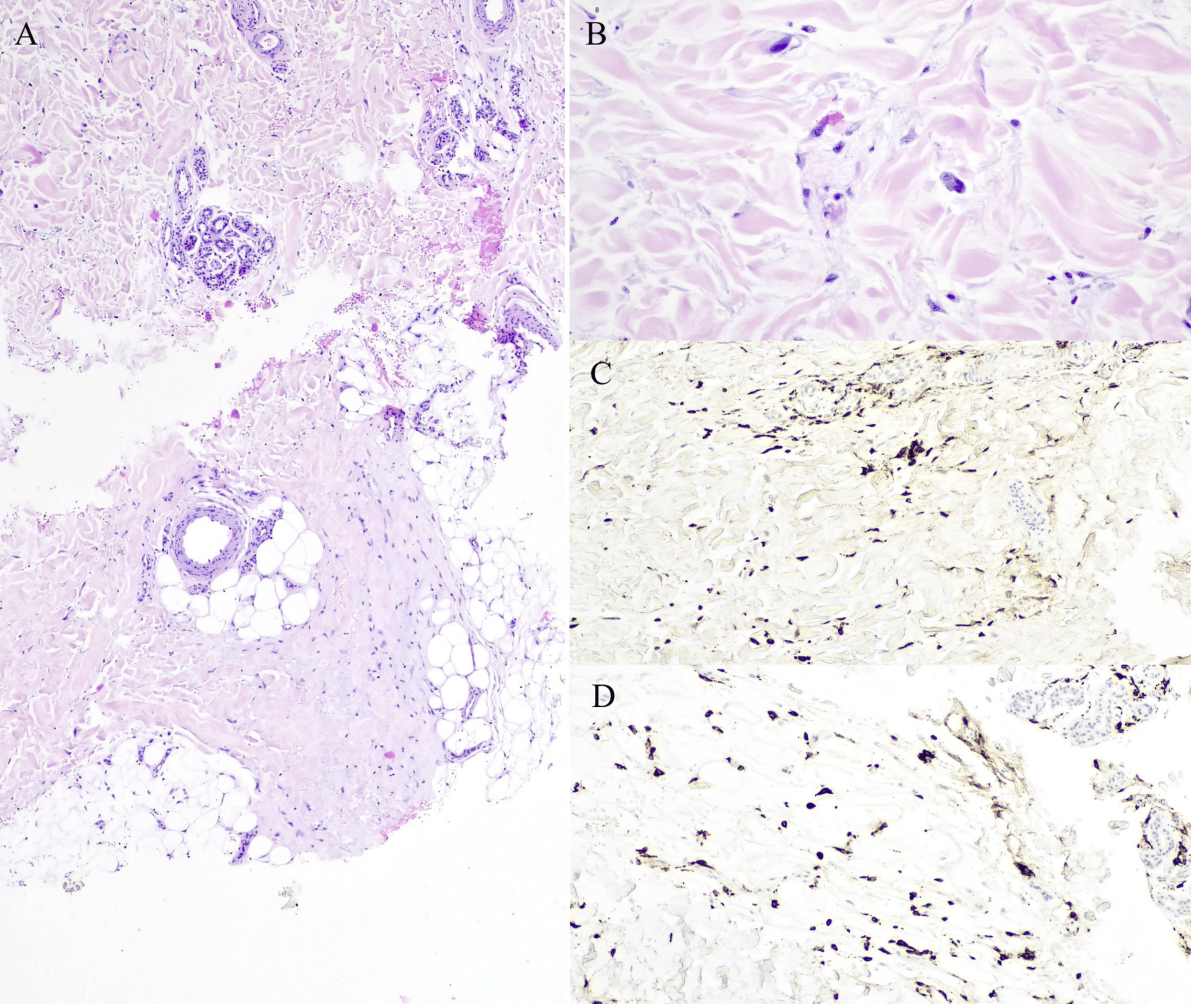
Dermal infiltrate constituted by abundant histocytes, spindle cells, and sparse mastocytes ((A) - hematoxylin and eosin, 40x). Reticular dermis with thickened collagen fibers with an eosinophilic and amorphous hue and fragmented aggregates of elastic fibers ((B) - hematoxylin and eosin, 400x). Immunohistochemistry with CD68 (C) and CD163 (D) positive histiocytes

Histological examination showed slight acanthosis and dermal infiltrate constituted by abundant histocytes, spindle cells, and sparse mastocytes. The mid and deep reticular dermis revealed thickened collagen fibers with an eosinophilic and amorphous hue and fragmented aggregates of elastic fibers (Figures 2A, 2B). Immunohistochemistry revealed mostly CD68 and CD163 positive histiocytes AE1/AE3, CK7, CAM5.2, and CD34 markers were negative (Figures 2C, 2D).

These findings confirmed the diagnosis of cutaneous sclerosis secondary to nab-paclitaxel. Dexamethasone 4 mg daily was started, and it was decided to resume chemotherapy with gemcitabine monotherapy. However, after three months, worsening sclerosis and marked edema led to suspension of treatment. The NALIRIFOX regimen (with liposomal irinotecan, fluorouracil, leucovorin, and oxaliplatin) was proposed as second-line therapy but was refused by the patient. During 20 months of follow-up and support therapy only, a slight improvement in the cutaneous sclerosis was noted, at which time the patient died due to disease burden.

## Discussion

Combined nab-paclitaxel and gemcitabine have emerged as one of the first-line treatments for metastatic pancreatic adenocarcinoma; however, it is associated with a high incidence of adverse reactions, most commonly neutropenia, anemia, and peripheral neuropathy [2]. However, only one case of cutaneous sclerosis associated with nab-paclitaxel and gemcitabine has been previously reported in the literature [1]. More reports of cutaneous sclerosis associated with paclitaxel, in continued broad use for decades, have been reported [3-7]. The physiopathology of these changes is still undefined. Most involved patients had been subjected to combination therapy involving multiple agents, including paclitaxel, gemcitabine, cisplatin, and/or carboplatin [1,3-7].

Clinical features include cutaneous sclerosis and edema, most prominent in the lower limbs, usually starting weeks to a few months after initiation of therapy, but with some rare cases of later onset, as in our case. Most patients did not present with systemic involvement or Raynaud phenomena. Antinuclear antibody positivity has been described in some patients, but with an unspecific speckled pattern and no association of scleroderma-specific antibodies (Scl-ab) [1].

Definitive diagnosis usually requires histological examination showing thickened dermal collagen, sclerosis, and sparse cellular infiltrate, with similarities with late classic scleroderma. Treatment approaches lack consistent evidence, with topical and/or systemic corticotherapy, methotrexate, or other immunosuppressants and phototherapy being used with variable results, which results in a chronic disease course with high morbidity [1]. Sclerosis is often progressive despite suspension of the offending agents [1,4].

## Conclusions

Cutaneous sclerosis secondary to chemotherapy with nab-paclitaxel and gemcitabine is a rare but serious adverse event that can lead to early suspension of treatment.
